# Detection of hemodynamic changes in a porcine lipopolysaccharide model of systemic inflammation using dynamic light scattering measurements of the microcirculation

**DOI:** 10.3389/fmed.2025.1522630

**Published:** 2025-02-21

**Authors:** Louwrina H. te Nijenhuis, Norani H. Gangaram-Panday, Patricia A. C. Specht, Ilya Fine, Nimrod Elstein, Egbert G. Mik, Floor A. Harms, Irwin K. M. Reiss, Willem van Weteringen

**Affiliations:** ^1^Department of Neonatal and Pediatric Intensive Care, Division of Neonatology, Erasmus MC Sophia Children’s Hospital, University Medical Center Rotterdam, Rotterdam, Netherlands; ^2^Department of Anesthesiology, University Medical Center Rotterdam, Rotterdam, Netherlands; ^3^Elfi-Tech Ltd., Rehovot, Israel

**Keywords:** lipopolysaccharide (endotoxin), microcirculation (skin), dynamic light scattering (DLS), animal model, sepsis-diagnostics

## Abstract

**Background:**

The microcirculation is affected during sepsis, yet there is currently no clinically available technology for sepsis detection in the microcirculation. This study aimed to detect microcirculatory changes using a dynamic light scattering (DLS) skin sensor during an endotoxic shock with a systemic inflammatory response in a porcine lipopolysaccharide (LPS) model.

**Methods:**

Thirty female Yorkshire x Norwegian Landrace pigs were divided into three groups: control, LPS, and LPS with resuscitation. After baseline measurements, LPS (1.75 μg∙kg^−1^∙h^−1^) was administered in progressively increasing dosages in the LPS and resuscitation groups. Two mDLS™ sensors, placed centrally and peripherally, measured total blood flow (TBF), relative blood velocity (RBV), and relative hemodynamic indices (relHIs) 1 h before (T0) and 1, 2, and 3 h after LPS administration (T1, T2, and T3). New DLS parameters describing heart rate variability (high-and low-frequency components HF and LF) and self-similarity (the Hurst exponent) were calculated.

**Results:**

No differences in TBF, RBV, and HF values were seen between the study groups after LPS administration. LF was peripherally higher at T2 in subjects receiving LPS than in controls. RelHIs showed a change in blood distribution between T0 and T1 in the resuscitation group. Both intervention groups showed a Hurst exponent decrease centrally at T2 and peripherally already at T1.

**Conclusion:**

Changes in microcirculatory parameters, relHIs, and the Hurst exponent, were recorded for 3 h following LPS administration. The Hurst exponent was significantly lower in the LPS and LPS with resuscitation groups than in controls. Further clinical studies are required to determine the sensitivity and specificity of the non-invasive mDLS™ sensor for sepsis detection.

## 1 Introduction

Sepsis is a disproportional response of the body to infection, which can result in a life-threatening organ dysfunction (1) and places a large burden on global healthcare systems (2, 3). Urgent recognition and early start of therapy are required to improve outcomes. The heterogeneity of the body’s response during sepsis impedes the search for a sensitive technology for diagnosing this systemic disease.

During sepsis, hemodynamic coherence between the macrocirculation and microcirculation is lost (4). Hypoperfusion, increased blood flow heterogeneity, and decreased skeletal muscle blood flow have been identified in the septic microcirculation (5–7). Microcirculatory deterioration during sepsis was shown to predict mortality more adequately than macrocirculatory parameters, such as arterial blood pressure and cardiac output (8). Technologies for monitoring the microcirculatory status are not yet incorporated into clinical practice as these are either not designed for the clinical setting or cannot measure continuously (9, 10).

Dynamic light scattering (DLS) technology can non-invasively measure microcirculatory parameters using a small skin sensor (11). The mDLS™ sensor emits near-infrared laser light, which is scattered back by moving erythrocytes. This scattered light is detected as a speckle pattern, which is a superposition of signals originating from different vessels and varies over time due to erythrocyte movement. Skin blood flow parameters can be derived from the fluctuations in speckle signal intensity. In recent studies, several parameters obtained from DLS technology have been evaluated: heart rate (HR), relative blood velocity (RBV), total blood flow (TBF), and relative hemodynamic indices (relHIs) (11–13). TBF represents the skin perfusion and is dependent on the total blood volume, while RBV represents the skin blood flow velocity independent of blood volume. RelHIs show the relative distribution of arterial and capillary blood in the microcirculation vasculature. RelHI1 represents the smallest vessels, while relHI5 represents the largest vessels (11).

Its non-invasiveness, small size, and multiplicity of available microcirculatory parameters make the mDLS™ sensor of interest for the assessment of microcirculatory changes during sepsis in the clinical setting. As sepsis is a multifactorial and complex disease with a large variety of symptoms, the first step is to validate the ability of this sensor to detect a lipopolysaccharide (LPS)-induced endotoxic shock in an animal model (14). The anatomy and physiology of pigs and their immunological response are similar to that of humans (15), providing an easy translation to human signals and clinical use.

The primary aim of this study is to evaluate the ability of DLS parameters to detect microcirculatory changes caused by LPS in an animal model. Current DLS parameters are evaluated, and new parameters are derived. The secondary objectives are to investigate differences between centrally and peripherally measured DLS parameters and changes in macrocirculatory parameters. Due to known microcirculatory hypoperfusion at sepsis onset, it is hypothesized that microcirculatory flow will decrease, starting peripherally.

## 2 Materials and methods

### 2.1 Study settings

In this laboratory investigation, DLS technology was applied in an LPS-based systemic inflammation model with female Yorkshire x Norwegian Landrace pigs (24–34 kg). With continuous infusion of LPS, a stable cell membrane component of Gram-negative bacteria, an endotoxic shock with a hemodynamic response similar to the human sepsis response is provoked in pigs (14). This proven model was chosen due to model experience and the ability to keep the animals stable for several hours. This study was part of a larger animal study, of which the protocol was approved on 15 September 2021 by the Central Authority for Scientific Procedures on Animals (license number AVD101002115658). The experiments were conducted in accordance with the Dutch Experiments on Animals Act and the ARRIVE guidelines (16).

### 2.2 Animal preparation

Animals were housed in pairs with environmental enrichment and given free access to food and water. They were acclimatized for at least 7 days. After overnight fasting with water *ad libitum*, the pigs were sedated by intramuscular injection of a mixture of tiletamine/zolazepam (6/6 mg/kg), xylazine (2 mg/kg), and atropine sulfate (0.5 mg/animal). After 10 min, consciousness was assessed by the corneal reflex, and the pigs were placed in a supine position. Anesthesia was induced by intravenous administration of ketamine (100–300 mg/animal) and tiletamine/zolazepam (50–100 mg/animal) in the auricular vein. All animals received a bolus of 500 mL colloid hydroxyethyl starch (Voluven®, Fresenius Kabi AG, Bad Homburg, Germany) at the start of the experiments.

The pigs were intubated using cuffed endotracheal tubes. During the entire experiment, the pigs were mechanically ventilated using pressure control ventilation (Maquet Servo-i Ventilator, Getinge AB, Rastatt, Germany). Ventilation settings (positive end-expiratory pressure, fraction of inspired oxygen, and breathing frequency) were set to maintain an end-tidal carbon dioxide range between 4 and 6 kPa and an arterial oxygen tension range between 80 and 120 mmHg.

After intubation, catheters were placed in the left femoral artery and vein using the Seldinger technique for arterial blood pressure monitoring, blood sampling, and medication administration. A thermodilution catheter was placed in the right femoral artery and jugular vein for cardiac output monitoring using the PiCCO_2_ technology (Getinge AB, Gothenburg, Sweden). After these preparation steps, the pigs were moved to the left lateral recumbent position and remained in this position for the duration of the experiment. Incisions were made for suprapubic cystostomy urine sampling and to provide access to the heart, liver, intestines, and kidneys as part of the main study protocol.

### 2.3 Animal maintenance

During the continuation of the experiment, the pigs were anesthetized and sedated by continuous infusion of midazolam (1.5 mg∙kg^−1^∙h^−1^), sufentanil (4 mg∙kg^−1^∙h^−1^), ketamine (5 mg∙kg^−1^∙h^−1^), and rocuronium bromide (4 mg∙kg^−1^∙h^−1^). The depth of sleep and signs of pain were continuously monitored during the experiment as humane endpoints, and anesthesia and analgesia were adjusted accordingly. If humane endpoints could not be resolved, the experiment was terminated immediately.

Norepinephrine (0.01–1.80 μg∙kg^−1^∙min^−1^), Sterofundin® ISO (3–30 mL∙kg^−1^∙h^−1^, B. Braun SE, Melsungen, Germany), and saline 0.9% (2–40 mL∙kg^−1^∙h^−1^) were continuously infused with administration rate adjustments to maintain mean arterial pressure (MAP), cardiac output, and filling state. The filling state was assessed based on pulse pressure variation calculated by the PiCCO_2_ device. Hypovolemia was treated when the pulse pressure variation was higher than 12%. Additional medication was used in case of events: amiodarone (50 mg/mL), epinephrine (1 mg/mL), lidocaine hydrochloride (10 mg/mL), calcium gluconate 10% (100 mg/mL), and metoprolol tartrate (1 mg/mL). Potassium chloride 15% (5–10 mL) was administered if potassium decreased below 3.5 mmol/L. All pigs received cefazolin (1,000 mg/animal) at the start and after 4 h to prevent Gram-positive infections during the experiment. A 1000 mg dose of magnesium sulfate was added to the first administration of Sterofundin® to prevent arrhythmias.

Normothermia (38–40°C) was maintained by positioning the pigs on a heating pad, using a heating blanket (3M™ Bair Hugger™ system, Saint Paul, Minnesota, United States), and administering Sterofundin® and saline at body temperature.

### 2.4 Study protocol

**Figure 1:**
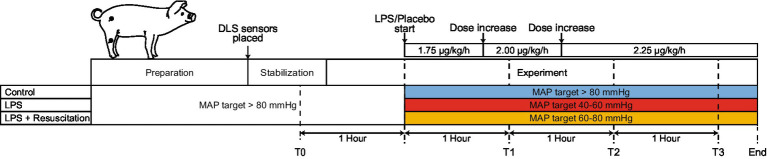
Schematic timeline of the different study phases. After a preparation and stabilization phase, measurements were performed, and LPS or a placebo (saline) was progressively administered. After the start of the LPS or placebo administration, therapeutic MAP ranges differed between the three study groups. Two DLS sensors were placed centrally and peripherally on the pig (black dots). LPS, lipopolysaccharide; DLS, dynamic light scattering; T, timepoint; MAP, mean arterial pressure.

Animals were consecutively assigned to three study groups (control, LPS, and LPS with resuscitation). After a stabilization period of at least 20 min and baseline measurements, an endotoxic shock with a systemic inflammation was induced in the LPS and LPS with resuscitation groups by intravenous administration of LPS (*Escherichia Coli* O127:B8, L3880, Sigma-Aldrich, Saint-Louis, Missouri, United States) in 0.9% NaCl solution. The LPS dose was started at 1.75 μg∙kg^−1^∙h^−1^, increased to 2.00 μg∙kg^−1^∙h^−1^ after 45 min, and 2.25 μg∙kg^−1^∙h^−1^ after another 45 min (Figure 1). The control group received saline (0.9% NaCl solution) as a placebo instead of LPS at the same volume and timing.

The three study groups differed in the received treatment to maintain MAP and glucose levels (Figure 1). MAP was maintained in specific ranges using norepinephrine and crystalloids, as crystalloids are the fluids of choice for resuscitation during sepsis (17, 18). Before LPS infusion, the MAP was maintained above 80 mmHg in all study groups. After LPS infusion, different desired MAP ranges were used for each study group. In the LPS group, the MAP was maintained between 40 and 60 mmHg. Pigs in the resuscitation group were resuscitated to maintain the MAP between 60 and 80 mmHg. In the control group, the MAP was maintained above 80 mmHg. Glucose was corrected in the control and resuscitation groups by administering 50% glucose (1 g∙kg^−1^∙h^−1^) if the glucose level dropped below 3.7 mmol/L (19).

Measurements were performed in the hour before LPS administration (T0) and 1, 2, and 3 h after LPS administration (T1, T2, and T3). Urine output and arterial and venous blood gases (including hematocrit, lactate, glucose, and potassium) were determined (ABL800 FLEX, Radiometer, Copenhagen, Denmark). Temperature, oxygen saturation, HR, and end-tidal carbon dioxide were continuously measured using a nasal temperature probe, pulse oximeter at the ear, 3-lead electrocardiogram, and capnography, respectively. Data were continuously and digitally logged with a sampling frequency of 1 Hz using a Siemens SC 9000XL Monitor (Siemens-Elema, Solna, Sweden). MAP, central venous pressure, and cardiac output were continuously recorded from an arterial line and the PiCCO_2_ device at 1 Hz. At the end of the experiment, the animals were terminated with potassium chloride. Pigs were *a priori* excluded from analysis if (1) baseline conditions were poor, (2) no systemic inflammatory response was obtained in pigs receiving LPS, (3) a systemic inflammatory response occurred in control pigs, (4) complications of surgery caused shock, or (5) the pig died before the end of the experiment (T3). Excluded animals were replaced.

### 2.5 DLS measurements

Continuous monitoring using two mDLS™ sensors (Elfi-Tech Ltd., Rehovot, Israel) was started after animal preparation by placing the sensors centrally (right thigh) and peripherally (right hind leg) on shaved skin using customized adhesive rings (Figure 1). The raw DLS signal was continuously logged with a sampling frequency of 100 Hz (DL_GUI version 1.7, Elfi-Tech Ltd., Rehovot, Israel). TBF and RBV were derived from the signal with a sampling frequency of 1 Hz, as previously described (11–13). RBV and TBF were filtered using a moving median filter (30 s window size) and were presented in arbitrary units (AU).

Five different relHIs were derived from the DLS blood flow signal by applying specific frequency bands on the DLS signal and dividing it by the total signal to prevent any variation between subjects due to sensor location and skin proximity. The frequency bands used to obtain the five relHIs were 0.5–1,000 Hz, 1,000–2000 Hz, 2000–4,000 Hz, 4,000–10,000 Hz, and 10,000–15,000 Hz. Each frequency band corresponds to a shear rate range, which is influenced by viscosity, blood flow, vessel type, and vascular resistance and can be used to estimate the blood distribution per vessel size (12). RelHIs were obtained with a sampling frequency of 100 Hz and were unitless as they represent relative values.

### 2.6 New DLS parameters

New parameters were derived from the DLS signal and investigated. The normalized low-frequency (LF) and high-frequency (HF) components of the heart rate variability from the DLS signal were determined, similar to these components found in electrocardiograms. The heart rate variability of the DLS blood flow signal was calculated from the pulsatile component of the blood flow signal. LF and HF are defined as the relative power of a specific frequency interval (0.04–0.15 Hz for LF and 0.15–0.40 Hz for HF) divided by the power of the blood flow signal in the heart rate variability spectrum (0.005–0.400 Hz). LF and HF are relative values and thus unitless. LF represents both sympathetic and parasympathetic behavior, while HF describes solely parasympathetic modulation (20, 21).

The Hurst exponent was calculated *post-hoc* from the blood flow signal to quantify its complexity and self-similarity over time. This exponent indicates the tendency of a time series signal to regress to the mean, follow a trend, or behave randomly. It is equal to 1 if the signal is completely self-similar, a value below 0.5 implies long-range anti-correlation over time, and exactly 0.5 suggests no correlation; the blood flow signal is only caused by Brownian motion (22, 23). Using a 180-s moving window with 90-s steps, the Hurst exponent was analyzed from fluctuations in blood flow, including the pulsatile alternating current (AC) and non-pulsatile direct current (DC) components. The AC and DC components of the Hurst exponent were obtained by applying a second-order high-pass and low-pass Butterworth filter with a cutoff frequency of 0.5 Hz on the DLS signal. The Hurst exponent was represented in AU. An overview of all DLS parameters used in this study is shown in Supplementary Figure 1.

### 2.7 Statistical analysis

Median values of all continuous parameters were determined in a window of 15 min before each timepoint (T0, T1, T2, and T3) for each pig. Between-group differences were analyzed at each timepoint using the Kruskal–Wallis test for multiple comparisons. Significant differences were further investigated using a Wilcoxon rank-sum test for pairwise comparison with Bonferroni correction for multiple testing. For each timepoint, the mean of all cutoff values with the maximal Youden’s index values was used to determine the sensitivity and specificity of DLS parameters to discriminate pigs in the LPS and control groups. The Friedman test was performed to investigate within-group differences over time. Significant differences were investigated using a Wilcoxon signed-rank test for pairwise comparison with Bonferroni correction. Central and peripheral measurements were compared using the Wilcoxon signed-rank test. Data were presented as median [interquartile range (IQR)], and a *p*-value <0.05 was considered significant. Analyses were performed using MATLAB (version R2022b, The MathWorks, Inc., Natick, MA, United States) and R (version 4.2.3, Inc., Boston, MA, United States). The sample size (*n* = 10 for each group) of this study was based on the calculation for the primary outcome in the main investigation.

## 3 Results

### 3.1 Study characteristics

In total, 35 pigs were measured in this study, of which 5 were excluded from analysis (1 died prematurely, 1 showed a poor condition before baseline, and 3 did not meet the assigned group criteria), resulting in 10 pigs per group. The included pigs had a median weight of 29.5 [28.2–31.4] kg; no differences were recorded between the groups. The total volume of crystalloids administered during the experiments did not differ between the groups (4,500 [3500–5,650] mL for controls, 5,500 [5000–6,875] mL for the LPS, and 5,750 [5050–6,750] mL for the resuscitation groups). The norepinephrine dosage did not differ between the groups for each timepoint (0.08 [0.08–0.15] μg∙kg^−1^∙min^−1^ for controls, 0.05 [0.01–0.14] μg∙kg^−1^∙min^−1^ in the LPS group, and 0.19 [0.15–0.41] μg∙kg^−1^∙min^−1^ in the resuscitation group at T3).

**Figure 2:**
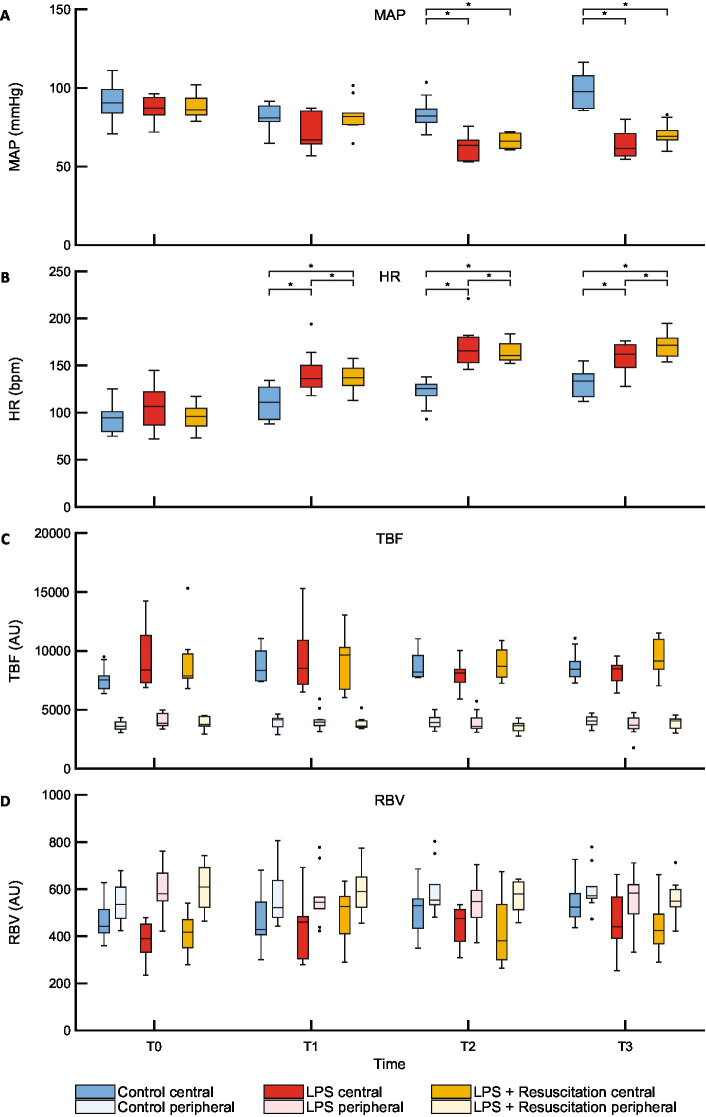
Boxplots of **(A)** MAP, **(B)** HR and DLS parameters **(C)** TBF, and **(D)** RBV. MAP, mean arterial pressure; HR, heart rate; DLS, dynamic light scattering; TBF, total blood flow; RBV, relative blood velocity; LPS, lipopolysaccharide; T, timepoint; bpm, beats per minute; AU, arbitrary unit. *p < 0.05 between the groups.

The boxplots in Figure 2A show a lower MAP at T2 and T3, whereas those in Figure 2B show a higher HR at T1, T2, and T3 in the intervention groups than in the control group. Hematocrit and lactate levels were similar at T0; however, at T1, hematocrit was higher in the LPS and resuscitation groups than in the control group (Supplementary Table S1). Lactate levels were higher in the LPS group than in the control and resuscitation groups after LPS administration.

### 3.2 TBF and RBV

TBF and RBV did not show any differences between the study groups at any timepoint (Figures 2C,D; Supplementary Table S1). However, in all pigs, TBF was significantly higher centrally (7,832 [7346–9,438] AU) than peripherally (3,766 [3539–4,237] AU, *p* < 0.01) at T0, whereas central RBV (426 [366–471] AU) was significantly lower than peripheral RBV (572 [517–675] AU, *p* < 0.01). Significant differences remained present during the course of the experiments (Table 1).

**Table 1:** Total blood flow and relative blood velocity measured using the mDLS™ sensor centrally and peripherally.

Parameter	Timepoint	Central (*n* = 30)	Peripheral (*n* = 30)	*p*-value
TBF (AU)	T0	7,832 [7346–9,438]	3,766 [3539–4,237]	<0.01
	T1	8,564 [7384–9,990]	3,960 [3567–4,183]	<0.01
	T2	8,147 [7790–9,269]	3,668 [3430–4,215]	<0.01
	T3	8,526 [7810–9,288]	3,939 [3592–4,285]	<0.01
RBV (AU)	T0	426 [366–471]	572 [517–675]	<0.01
	T1	470 [408–546]	544 [505–645]	<0.01
	T2	475 [376–535]	556 [514–614]	<0.01
	T3	477 [408–566]	566 [535–611]	0.01

Data represent the pooled data of all study groups (control, LPS, and LPS with resuscitation) together. Data are presented as median [interquartile range]. The *p*-values represent the comparison between the central and peripheral measurements using a Wilcoxon signed-rank test. TBF, total blood flow; RBV, relative blood velocity; T, timepoint; AU arbitrary unit; LPS, lipopolysaccharide.

### 3.3 RelHIs

**Figure 3:**
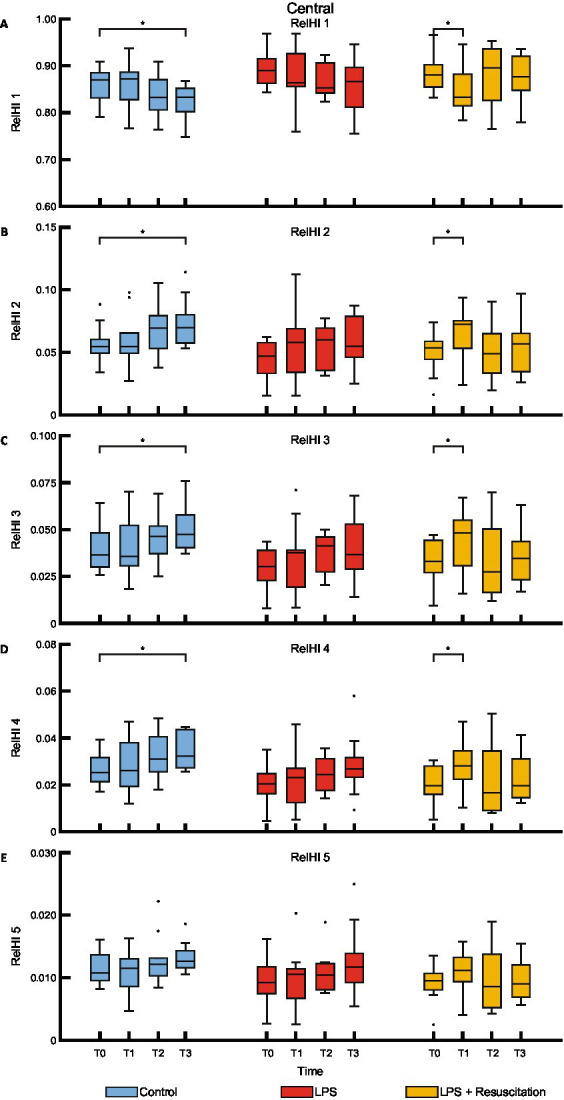
Boxplots of the five relHIs **(A–E)** measured with the centrally placed DLS sensor. RelHIs, relative hemodynamic indices; DLS, dynamic light scattering; LPS, lipopolysaccharide; T, timepoint. *p < 0.05 between the groups.

**Figure 4:**
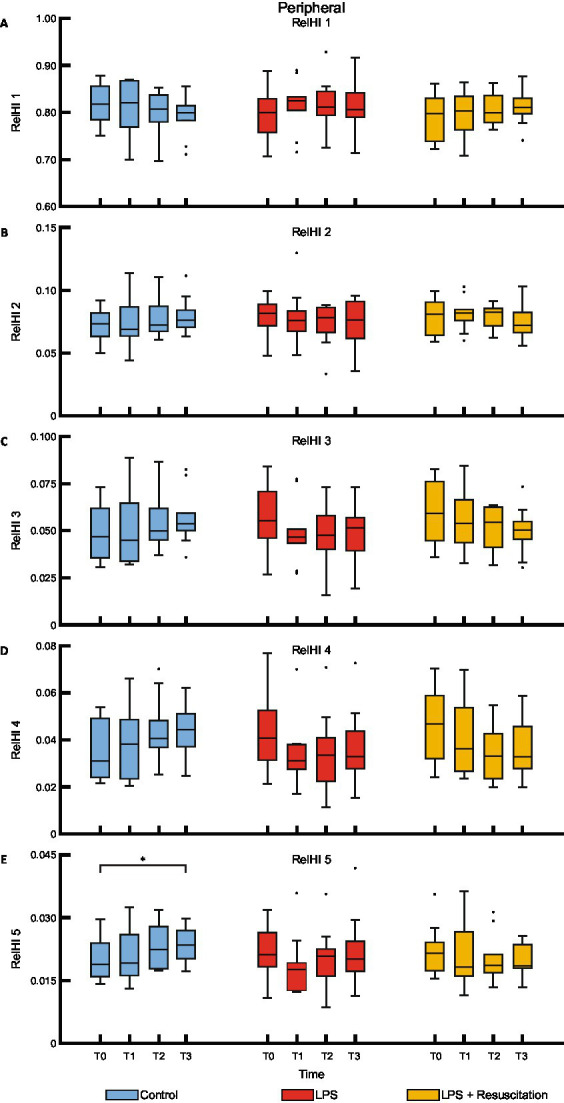
Boxplots of the five relHIs **(A–E)** measured with the peripherally placed DLS sensor. RelHIs, relative hemodynamic indices; DLS, dynamic light scattering; LPS, lipopolysaccharide; T, timepoint. *p < 0.05 between the groups.

The relHIs showed changes in blood distribution over time; no visual differences were present between the groups (Figure 3). Within the resuscitation group, significant differences were present between T0 and T1 in the centrally measured relHIs; the other groups did not differ between these timepoints. The resuscitation group showed a significant decrease in relHI1 (0.880 [0.860–0.898] at T0 to 0.833 [0.816–0.877] at T1, *p* = 0.02), while relHI2, relHI3, and relHI4 increased significantly from 0.054 [0.046–0.059] to 0.072 [0.056–0.075] (*p* = 0.02), from 0.033 [0.028–0.042] to 0.048 [0.033–0.054] (*p* = 0.02), and from 0.020 [0.017–0.027] to 0.028 [0.022–0.034] (*p* = 0.02), respectively. In the controls, significant differences were present between T0 and T3. Peripherally measured relHIs only showed changes over time in the controls, showing a difference between T0 and T3 (Figure 4).

### 3.4 New DLS parameters

**Figure 5:**
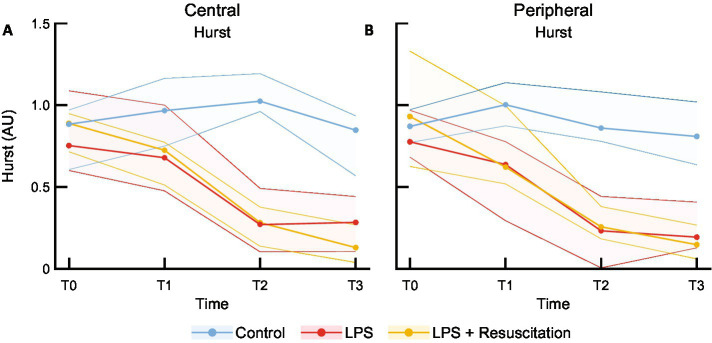
Median and interquartile ranges of Hurst exponent measured with the **(A)** central and **(B)** peripheral DLS sensors. DLS, dynamic light scattering; LPS, lipopolysaccharide; T, timepoint; AU, arbitrary unit.

HF showed no significant differences between the study groups (Supplementary Table S1). LF was peripherally significantly higher in the intervention groups at T2 than in the controls. Figure 5A shows a decrease in the centrally measured Hurst exponent after LPS administration in both the LPS (0.75 [0.63–1.07] at T0 to 0.27 [0.12–0.46] AU at T2, *p* = 0.01) and resuscitation groups (0.89 [0.72–0.95] to 0.28 [0.14–0.37] AU, *p* = 0.01), which were significantly different from the control group at T2 (1.03 [0.96–1.18] AU, *p* < 0.01) and T3 (Supplementary Table S2). The Hurst exponent measured peripherally (Figure 5B) already showed significant differences at T1 between the control (1.00 [0.89–1.14] AU) and both the LPS (0.64 [0.38–0.77] AU, *p* = 0.03) and resuscitation groups (0.62 [0.53–0.95] AU, *p* = 0.01). At T2, cutoff values of 0.72 AU and 0.69 AU for central and peripheral measurements were found, respectively, indicating a sensitivity and specificity of 100% between the control and LPS groups. Decreases in Hurst were mainly caused by the AC component (Supplementary Figure 2), showing significant differences between the groups at T1, T2, and T3 peripherally and at T2 and T3 centrally, while no differences were observed in the DC component (Supplementary Table S2).

## 4 Discussion

This is the first study evaluating the ability of a non-invasive DLS sensor to detect microcirculatory changes caused by LPS in an animal model. No differences were recorded between the study groups in TBF, RBV, and HF levels following LPS administration. However, LF was higher peripherally in pigs receiving LPS than in the controls. Within the resuscitation group, changes in relHIs were observed over time. The Hurst exponent demonstrated a clear distinction between the control subjects and subjects receiving LPS, which was first visible peripherally.

The LPS model is a simple and reproducible model, inducing a hemodynamic response mimicking the systemic inflammatory response in humans by intravenous administration of LPS (24). Pigs have shown a hemodynamic response similar to humans (14, 25), as demonstrated by the MAP reduction and HR and lactate increases in the intervention groups of this study, suggesting an endotoxic shock with a systemic inflammation following LPS administration. The usability of the LPS model in animals is debated in literature (14, 26–28), as not all animals turned out to be endotoxin sensitive or showed opposite symptoms, and other components of bacteria might be of similar importance to induce the systemic inflammatory response.

The microcirculatory DLS parameters, RBV and TBF, remained constant across all study groups. We expected TBF and RBV to reduce after LPS infusion, as capillary blood is known to be altered during sepsis (29). Other technologies measuring microcirculatory blood flow such as laser Doppler flowmetry and video microscopy did report changes in flow parameters in sepsis models. Previous research using live *Escherichia coli* and *Neisseria meningitidis* as sepsis models showed reduced laser Doppler tissue perfusion and capillary flow velocities in the microcirculation of septic pigs (30, 31). Orthogonal polarization spectroscopy showed a reduced erythrocyte velocity after administration of *Escherichia coli* in the study of Verdant et al. (32). The conflicting results in literature concerning both RBV and TBF can be explained by the use of different microcirculatory flow technologies and their measurement depths (33, 34). The DLS sensor uses erythrocyte scattering intensity to determine blood flow and velocity, whereas other technologies are based on blood absorption and Doppler shift, and use light at different wavelengths. Differences in blood flow might be diminished in TBF by the higher hematocrit levels in the LPS group. The DLS measurement depth at the skin is approximately 1.3 mm; however, since light penetration depth depends on factors such as wavelength, the measurement depth may vary for other optical technologies. The differing observations could also be explained by the heterogeneous reaction of the microcirculation to sepsis (35). In addition, other studies used intravascular infusion of live bacteria, probably invoking a fiercer septic reaction, while our study induced an endotoxic shock with systemic inflammation. Our results suggest that although macrocirculatory changes were recorded, microcirculatory blood flow and velocity were preserved. The significant differences between the central and peripheral measured TBF and RBV in all study groups potentially reflect the influence of the higher central skin temperature on these parameters, as increased skin temperature causes vasodilation and increased skin blood flow (36).

The shift in relHIs from the smallest vessels to larger vessels after LPS administration in the resuscitation group can be related to capillary blood redistribution, as is also noticed during sepsis (29, 37). Increased capillary blood flow heterogeneity, along with a reduced proportion of perfused vessels and a reduced perfused capillary density, have been observed in pigs receiving *Escherichia coli* and LPS (32, 38). However, as the LPS group in our study did not show relHI changes, these cannot be solely attributed to the LPS model. The administration of fluids and vasoactive agents to keep MAP in predefined ranges in the intervention groups also has an effect on blood distribution as an increased proportion of perfused vessels has been reported after resuscitation (38). Changes in relHIs in all groups might therefore be attributed to fluid management and administration of vasoactive agents or general deterioration at the end of the experiment.

The observed higher peripheral LF in the intervention groups is compliant with the results in the literature. Frequency domain parameters of heart rate variability measurements of electrocardiograms showed an elevated LF and LF/HF ratio in both septic humans and animals (39–41), suggesting a shift toward sympathetic nervous activity (20). No significant differences in HF were recorded in this study, probably due to differences in technology and its relation to physiological activity and the severity of the inflammatory response.

The Hurst exponent is a non-linear parameter describing the long-term memory of a time series. This is the first time the Hurst exponent is calculated to describe the blood flow oscillations in the DLS signal and is investigated during systemic inflammation. Although TBF and RBV did not change, the decreased Hurst exponent reflects changes in blood flow oscillations due to changes in vasomotion after LPS administration. Vasomotion concerns the oscillation in blood vessel wall tone over time, causing flowmotion (oscillations in blood flow) (42). Previous research has also shown increased skin blood flow oscillations using laser Doppler in septic patients, reflecting locally regulated changes in peripheral vascular tone (43). The observed early peripheral Hurst exponent decrease might reflect the response of the body to maintain blood supply to protect vital organs during inflammation. The decrease in the Hurst exponent is mainly attributed to changes in the AC component, suggesting that non-linear blood flow behavior primarily changes in the pulsatile part of the signal. Both the LPS and resuscitation groups showed a decreased Hurst, which did not improve despite resuscitation and macrocirculatory improvement, making Hurst a parameter potentially reflecting microcirculatory changes without being influenced by fluid administration. In this study, the Hurst exponent was retrospectively determined after acquisition of the whole blood flow signal, using a 3-min window with steps of 90s. Real-time Hurst calculation is feasible and would allow for direct clinical observation of changes in blood flow oscillatory behavior, and warn for systemic inflammation or sepsis. It should be noted that the high sensitivities and specificities of the Hurst exponent for a clear delineation between the control and LPS groups were calculated with limited data. For more robust metrics, we recommend a larger sample size in future studies.

There are some noteworthy study limitations. Blinded group allocation was not possible as MAP targeted for fluid management differed between the three groups. In addition, pigs in both intervention groups received excessive fluid administration and vasoactive therapy to keep them alive until the end of the experiments. These therapies might mitigate the effect of LPS, resulting in the absence of any significant microcirculatory alterations, as reflected by the minor increase in lactate. This limitation could explain why some DLS parameters did not show any changes. The study setup makes it difficult to distinguish whether these observations are due to these small microcirculatory differences between the groups or due to the inability of the mDLS™ sensor. The use of more severely ill pigs and additional microcirculatory monitoring techniques within the same model may improve the evaluation of the mDLS™ sensor’s ability to detect microcirculatory alterations in an LPS model. Despite the possible mitigation of the microcirculatory changes due to the fluid administration, the Hurst parameter can still detect changes after LPS administration. Another limitation is that the microcirculation may be additionally affected by the performed surgical procedures. The elapsed time before the systemic inflammatory response and its severity differed between pigs despite the use of a homogeneous breed and the choice of LPS dose to induce a response within 60 min. This limitation can result in smaller parameter differences at T1; however, it is assumed that all pigs showed symptoms of systemic inflammation within 2 h after LPS administration. In addition, the response to LPS is investigated over 3 h, while several inflammatory symptoms such as edema develop at a later stage. As mentioned, caution is recommended in the translation from the LPS model to the human systemic inflammatory response and sepsis (27, 44). Although the specific LPS-induced systemic inflammation in the applied model did not result in the specific flow changes that were seen in sepsis models, a clear effect that was observed in the Hurst exponent indicates its potential applicability in human systemic inflammation and sepsis.

## 5 Conclusion

The findings of this study demonstrate the ability of the mDLS™ sensor to detect microcirculatory changes in an LPS model. The Hurst exponent was significantly lower in pigs receiving LPS, and relHIs showed a shift in blood distribution. Microcirculatory LF increased, but RBV, TBF, and HF did not change following LPS administration. Thus, monitoring microcirculation continuously using DLS technology holds the potential for early detection of systemic inflammation or even sepsis, as well as therapeutic implications.

## Data Availability

The original contributions presented in the study are included in the article/Supplementary material, further inquiries can be directed to the corresponding author.
