# Supplementary material for: Detection of hemodynamic changes in a porcine lipopolysaccharide model of systemic inflammation using dynamic light scattering measurements of the microcirculation
Source: Front Med (Lausanne). 2025 Feb 21;12:1522630. doi: 10.3389/fmed.2025.1522630 (PMC11885275; doi:10.3389/fmed.2025.1522630)
Supplement: Supplementary file 1 [file Data_Sheet_1.pdf]

Supplementary Material

Contents

1 **Supplemental Figure S1** Overview of DLS parameters 2

2 **Supplemental Table S1** Laboratory values, hemodynamic parameters and dynamic light scattering parameters per group 3

3 **Supplemental Table S2** Centrally and peripherally measured Hurst exponent and its alternating and direct current components per group 6

4 **Supplemental Figure S2** Centrally and peripherally measured alternating current and direct current components of Hurst exponent 8

1 **Supplemental Figure S1** Overview of DLS parameters

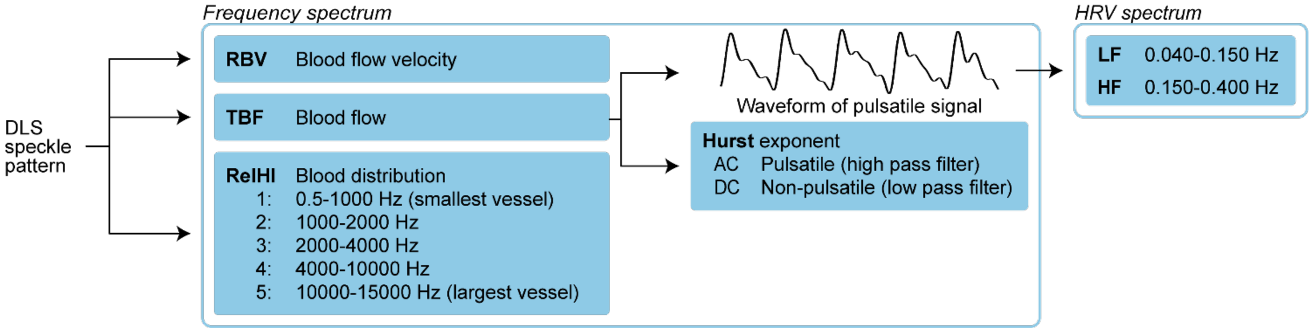

Figure S1 Overview of DLS parameters used in the study. DLS, dynamic light scattering; RBV, relative blood velocity; TBF, total blood flow; RelHI, relative hemodynamic index; AC, alternating current; DC, direct current; HRV, heart rate variability; LF, low frequency component; HF, high frequency component.

2 **Supplemental Table S1** Laboratory values, hemodynamic parameters and dynamic light scattering parameters per group

Parameter	Timepoint	Control (n = 10)	LPS (n = 10)	LPS with resuscitation (n = 10)	p-value
Ht (%) (venous)	T0	23.5 [21.2-24.5]	22.2 [20.8-23.1]	21.4 [20.8-22.6]	0.24
	T1	23.4 [20.3-24.6]	25.2 [25.0-26.8]	26.6 [24.9-27.2]	0.01ǂ
	T2	23.9 [20.9-25.3]	25.8 [25.0-26.4]	25.5 [24.3-26.8]	0.05
	T3	24.2 [22.0-25.9]	25.9 [24.7-26.6]	25.0 [23.7-26.4]	0.37
Lactate (mmol/L) (arterial)	T0	1.1 [0.9-1.3]	1.2 [1.0-1.6]	1.0 [0.8-1.4]	0.39
	T1	0.9 [0.7-1.1]	1.7 [1.2-2.7]	1.2 [1.0-1.2]	<0.01ǂ^
	T2	0.8 [0.7-0.9]	1.7 [1.5-2.2]	1.4 [1.3-1.5]	<0.01ǂ*
	T3	0.7 [0.6-0.8]	2.0 [1.8-2.7]	1.8 [1.6-2.0]	<0.01ǂ*
MAP (mmHg)	T0	91 [85-99]	87 [83-93]	86 [83-92]	0.55
	T1	81 [79-87]	67 [65-83]	82 [78-84]	0.15
	T2	82 [79-86]	63 [54-67]	66 [63-71]	<0.01ǂ*
	T3	98 [87-106]	62 [57-70]	69 [67-72]	<0.01ǂ*
HR (bpm)	T0	95 [81-101]	106 [90-119]	96 [87-104]	0.36
	T1	111 [93-125]	136 [129-149]	137 [129-147]	<0.01ǂ*
	T2	126 [118-130]	166 [155-180]	160 [157-170]	<0.01ǂ*
	T3	134 [120-140]	162 [148-171]	172 [160-178]	<0.01ǂ*

Supplemental Table S1 Continued

TBF central (AU)	T0	7533 [6968-7806]	8371 [7484-10766]	7876 [7725-9672]	0.13
	T1	8345 [7581-9819]	8524 [7247-10392]	9648 [6807-10172]	1.00
	T2	8204 [7855-9509]	8125 [7528-8358]	8691 [7803-9868]	0.49
	T3	8442 [7937-9012]	8469 [7526-8704]	9145 [8606-10955]	0.17
TBF peripheral (AU)	T0	3600 [3390-3946]	3856 [3676-4582]	3766 [3654-4331]	0.29
	T1	4148 [3651-4272]	3952 [3689-4144]	3619 [3551-4041]	0.56
	T2	3922 [3587-4320]	3589 [3430-4204]	3646 [3261-3815]	0.28
	T3	4048 [3751-4352]	3691 [3445-4260]	4054 [3527-4188]	0.57
RBV central (AU)	T0	442 [417-503]	390 [341-450]	417 [364-462]	0.23
	T1	429 [411-530]	460 [333-483]	526 [426-563]	0.67
	T2	530 [443-553]	475 [385-507]	381 [315-522]	0.17
	T3	524 [483-577]	440 [392-548]	424 [378-486]	0.09
RBV peripheral (AU)	T0	535 [483-596]	580 [555-649]	609 [534-688]	0.21
	T1	522 [481-620]	544 [519-564]	590 [526-650]	0.54
	T2	554 [536-608]	548 [493-594]	580 [514-620]	0.85
	T3	572 [563-606]	583 [508-616]	549 [527-587]	0.44
HF central	T0	0.82 [0.80-0.85]	0.63 [0.63-0.78]	0.76 [0.68-0.79]	0.18
	T1	0.63 [0.60 0.71]	0.62 [0.61-0.64]	0.62 [0.61-0.66]	0.90
	T2	0.65 [0.61 0.67]	0.65 [0.61-0.65]	0.64 [0.62-0.65]	0.82
	T3	0.64 [0.61-0.66]	0.64 [0.61-0.65]	0.62 [0.61-0.63]	0.36

Supplemental Table S1 Continued

HF peripheral	T0	0.81 [0.69-0.85]	0.62 [0.61-0.69]	0.76 [0.62-0.78]	0.08
	T1	0.65 [0.60-0.69]	0.62 [0.61-0.63]	0.62 [0.61-0.64]	0.59
	T2	0.65 [0.63-0.75]	0.63 [0.60-0.64]	0.63 [0.61-0.65]	0.13
	T3	0.63 [0.62-0.67]	0.64 [0.60-0.69]	0.62 [0.61-0.64]	0.84
LF central	T0	0.13 [0.11-0.16]	0.27 [0.16-0.27]	0.12 [0.11-0.14]	0.05^
	T1	0.24 [0.16-0.29]	0.31 [0.29-0.31]	0.28 [0.26-0.30]	0.06
	T2	0.27 [0.25-0.30]	0.28 [0.27-0.32]	0.29 [0.28-0.30]	0.35
	T3	0.27 [0.25-0.29]	0.27 [0.27-0.30]	0.30 [0.28-0.32]	0.20
LF peripheral	T0	0.10 [0.09-0.20]	0.26 [0.20-0.28]	0.13 [0.09-0.18]	0.02^
	T1	0.24 [0.14-0.26]	0.30 [0.29-0.31]	0.30 [0.29-0.30]	0.05
	T2	0.25 [0.14-0.26]	0.30 [0.27-0.32]	0.29 [0.29-0.31]	<0.01ǂ*
	T3	0.27 [0.22-0.28]	0.29 [0.24-0.31]	0.30 [0.29-0.31]	0.09

Data are presented as median [interquartile range]. LPS, lipopolysaccharide; Ht, hematocrit; MAP, mean arterial pressure; HR, heart rate; bpm, beats per minute; TBF, total blood flow; AU, arbitrary unit; RBV, relative blood velocity; HF, high-frequency component; LF, low-frequency component; T, timepoint.

ǂ Significant difference between control and LPS group (Bonferroni adjusted *p*-value)
* Significant difference between control and resuscitation group (Bonferroni adjusted *p*-value)
^ Significant difference between LPS and resuscitation group (Bonferroni adjusted *p*-value)

3 **Supplemental Table S2** Centrally and peripherally measured Hurst exponent and its alternating and direct current components per group

Parameter	Location	Timepoint	Control (n = 10)	LPS (n = 10)	LPS with resuscitation (n = 10)	p-value
Hurst (AU)	Central	T0	0.89 [0.64-0.97]	0.75 [0.63-1.07]	0.89 [0.72-0.95]	0.95
		T1	0.97 [0.80-1.13]	0.68 [0.48-0.98]	0.73 [0.54-0.77]	0.23
		T2	1.03 [0.96-1.18]	0.27 [0.12-0.46]	0.28 [0.14-0.37]	<0.01‡*
		T3	0.85 [0.61-0.93]	0.28 [0.12-0.41]	0.13 [0.06-0.25]	<0.01‡*
	Peripheral	T0	0.87 [0.78-0.97]	0.78 [0.70-0.96]	0.93 [0.67-1.31]	0.73
		T1	1.00 [0.89-1.14]	0.64 [0.38-0.77]	0.62 [0.53-0.95]	<0.01‡*
		T2	0.86 [0.78-1.04]	0.23 [0.03-0.43]	0.26 [0.18-0.37]	<0.01‡*
		T3	0.81 [0.65-0.99]	0.19 [0.13-0.40]	0.15 [0.06-0.25]	<0.01‡*
Hurst AC (AU)	Central	T0	0.88 [0.64-0.96]	0.75 [0.62-1.06]	0.88 [0.71-0.93]	0.98
		T1	0.96 [0.78-1.12]	0.68 [0.48-0.97]	0.72 [0.53-0.77]	0.25
		T2	1.02 [0.96-1.17]	0.27 [0.12-0.45]	0.28 [0.13-0.37]	<0.01‡*
		T3	0.85 [0.61-0.92]	0.28 [0.12-0.41]	0.13 [0.06-0.25]	<0.01‡*
	Peripheral	T0	0.86 [0.76-0.95]	0.77 [0.69-0.96]	0.91 [0.67-1.30]	0.79
		T1	0.99 [0.88-1.13]	0.63 [0.38-0.77]	0.62 [0.53-0.95]	<0.01‡*
		T2	0.86 [0.78-1.04]	0.23 [0.02-0.42]	0.25 [0.18-0.36]	<0.01‡*
		T3	0.81 [0.65-0.99]	0.19 [0.13-0.40]	0.14 [0.06-0.25]	<0.01‡*

Supplemental Table S2 Continued

Hurst DC (AU)	Central	T0	3.11 [3.01-3.38]	3.12 [2.94-3.27]	3.24 [3.06-3.38]	0.89
		T1	3.01 [2.63-3.08]	2.65 [2.24-3.01]	2.51 [2.14-2.89]	0.31
		T2	2.44 [2.19-2.91]	2.86 [2.35-3.35]	3.00 [2.61-3.40]	0.18
		T3	2.68 [2.44-2.99]	3.06 [2.46-3.49]	2.93 [2.86-3.21]	0.30
	Peripheral	T0	2.98 [2.71-3.21]	2.72 [2.25-3.33]	3.02 [2.73-3.24]	0.82
		T1	2.61 [2.44-2.99]	2.28 [2.08-2.90]	2.25 [2.11-2.53]	0.16
		T2	2.41 [2.13-2.57]	2.91 [2.20-3.48]	2.81 [2.70-3.17]	0.10
		T3	2.46 [2.31-2.61]	2.60 [2.28-3.41]	3.07 [2.72-3.32]	0.08

Data are presented as median [interquartile range]. LPS, lipopolysaccharide; AC, alternating current; DC, direct current; AU, arbitrary unit; T, timepoint.

ǂ Significant difference between control and LPS group (Bonferroni adjusted *p*-value)
* Significant difference between control and resuscitation group (Bonferroni adjusted *p*-value)

4 **Supplemental Figure S2** Centrally and peripherally measured alternating current and direct current components of Hurst exponent

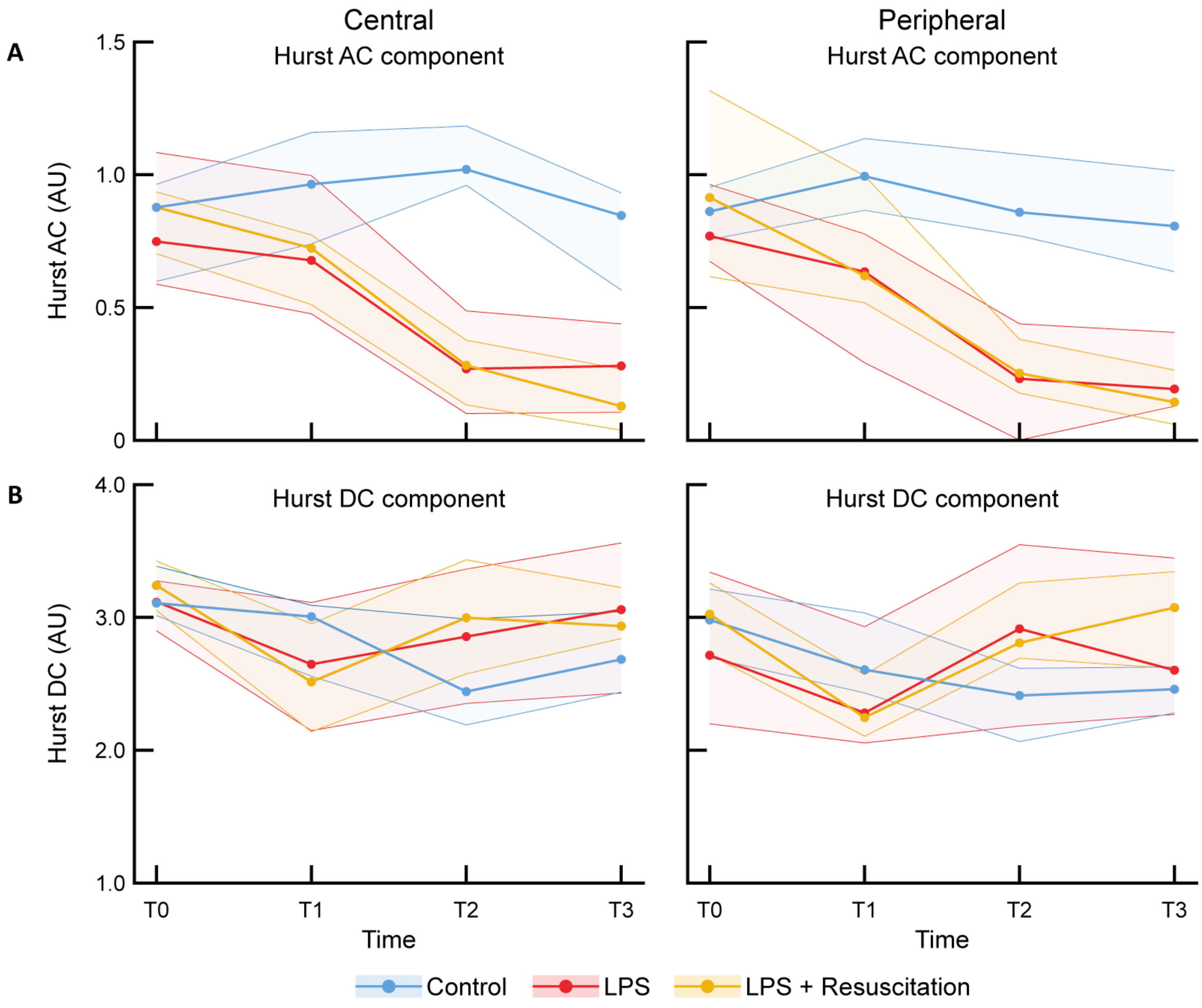

Figure S2 Median and interquartile ranges of the Hurst exponent. **(A)** Hurst exponent of the AC component and **(B)** of the DC component of the DLS signal, measured with the central and peripheral mDLS™ sensor for the three study groups. AC, alternating current; DC, direct current; DLS, dynamic light scattering; LPS, lipopolysaccharide; T, timepoint; AU arbitrary unit.
